# Draft genome sequence of a multidrug-resistant *Staphylococcus haemolyticus* MRSH_GSTU_NS23_BD strain isolated from a nurse’s hand swab in Bangladesh

**DOI:** 10.1128/mra.00609-26

**Published:** 2026-07-01

**Authors:** Abdul Malek, Sazzad Hossain Sagor, Md. Naim, Md. Yamun Hasan, Rabeya Khanam, Tanjim Ishraq Rahaman, Ismatara Munia, Raihan Shafi, Md. Sahabuddin

**Affiliations:** 1 Department of Biotechnology and Genetic Engineering, Faculty of Life Science, Gopalganj Science and Technology University 421965, Gopalganj, Bangladesh; 2 Biotechnology and Genetic Engineering Discipline, Life Science School, Khulna University, Khulna, Bangladesh; Fluxus Inc., Sunnyvale, California, USA

**Keywords:** multidrug resistance, *Staphylococcus haemolyticus*, coagulase-negative staphylococci, genomes, antibiotic resistance

## Abstract

Here, we report the draft genome sequence of *Staphylococcus haemolyticus* strain MRSH_GSTU_NS23_BD isolated from a nurse’s hand swab. The genome is 2,421,936 bp in length and contains 2,377 CDSs, 56 tRNAs, and three rRNAs. It harbors nine and 15 predicted virulence factors and antimicrobial resistance genes, respectively.

## ANNOUNCEMENT

*Staphylococcus haemolyticus* is the second most common coagulase-negative staphylococci (CoNS), accounting for 10–20% of clinical CoNS infections and causing several types of diseases, such as sepsis, otitis, bloodstream infections, chronic prostatitis, diabetic foot ulcers, and urinary tract infections (1, 2). Methicillin-resistant *S. haemolyticus* (MRSH) strains exhibit multiple resistance patterns and serve as a genetic reservoir of virulence and antimicrobial resistance (AMR) genes that can spread to other bacteria (3, 4). The widespread presence of those multidrug-resistant (MDR) bacteria can restrict the availability of antimicrobial agents, posing a concerning public health threat (5).

*Staphylococcus haemolyticus* MRSH_GSTU_NS23_BD has been isolated in hand swabs collected from nurses at Gopalganj Sadar Hospital, Gopalganj, Bangladesh in 2024. To summarize, 15 hand swab specimens were collected under aseptic conditions utilizing sterile cotton swabs and subsequently processed by primarily enriching in nutrient broth at 37°C for 24 h individually. Following a 10-fold serial dilution were performed in peptone water, and single pure colonies were successfully isolated on selective mannitol salt agar (MSA) media by incubating at 37°C overnight. *S. haemolyticus* was first identified by standard biochemical assays, such as catalase, oxidase, coagulase, and MR-VP, and Gram staining. Antimicrobial susceptibility testing was performed via the Kirby-Bauer disk diffusion technique according to the Clinical and Laboratory Standards Institute guidelines (6). Among the eight identified isolates, we performed whole-genome sequencing (WGS) on the multidrug-resistant *Staphylococcus haemolyticus* isolate MRSH_GSTU_NS23_BD to evaluate its antimicrobial resistance patterns, virulence profiles, and genomic characteristics. The isolate showed resistance against nine antibiotics, namely, cefoxitin, ceftazidime-tazobactam, azithromycin, erythromycin, ceftriaxone, moxifloxacin, co-trimoxazole, clindamycin, and nitrofurantoin, among 13 antibiotics.

For WGS, the *Staphylococcus haemolyticus* strain MRSH_GSTU_NS23_BD was cultured overnight on nutrient agar at 37°C. Genomic DNA was extracted using the Wizard Genomic DNA Purification Kit (Cat. A1620, Promega, Madison, WI, USA), and the DNA library was prepared using Illumina DNA Prep (Illumina, San Diego, CA, USA). The quality of the extracted DNA was assessed using a NanoDrop spectrophotometer, and library quality was evaluated by Agilent 2100 Bioanalyzer before sequencing. Sequencing was carried out to the Genome Center of the International Center for Diarrhoeal Disease Research, Bangladesh, on the Illumina NextSeq 2000 platform, generating Illumina paired-end raw 2 × 150 bp reads. The raw reads were quality controlled using FastQC v0.12.1 (7) and trimmed by fastp v0.23.4 (8). *De novo* assembly was performed using SPAdes v3.15.5 (9, 10) and classified using standard Kraken2 v2.1.3 (11) containing the National Center for Biotechnology Information (NCBI) RefSeq bacteria genome database. For accurate species-level classification, we used a confidence threshold of 0.1 to eliminate low-confidence queries. Genome completeness was evaluated by CheckM v1.2.5 (12).

The BV-BRC (13) and the NCBI Prokaryotic Genome Annotation Pipeline v.6.10 (14) were used to resolve a final genome of 88 contigs with a total length of 2,421,936 bp (*N*_50_, 73,303 bp; largest contig 178,454 bp) and a guanine-cytosine (GC) content of 32.57%. Analysis of the assembled contig with PathogenFinder v2.0 (15) showed the isolate had a 54.17% probability of being a human pathogen. Analysis with RGI v6.0.5 from the CARD v4.0.2 (16) found 15 AMR genes; all the software was run in default parameters unless otherwise specified. The genomic features of sequenced *S. haemolyticus* were presented in Table 1.

**TABLE 1:** Genomic features of *Staphylococcus haemolyticus* strain MRSH_GSTU_NS23_BD

Parameters	*Staphylococcus haemolyticus* strain MRSH_GSTU_NS23_BD
Contigs	88
Genome size (bp)	2,421,936 bp
GC content (%)	32.57
Contig L50	11
Contig *N*_50_	73,303
Genome completeness (%)	99.25%
Genome coverage	110.4×
Number of paired-end raw reads	1,275,305
Genes (total)	2,440
CDSs (total)	2,377
Genes (coding)	2,329
Total RNAs	63
No. of tRNAs	56
No. of rRNAs	3
No. of ncRNAs	4
No. of pseudo genes (total)	48
No. of hypothetical proteins	393
Proteins with functional assignments	2,011
Proteins with EC number assignments	702
Proteins with GO assignments	579
Proteins with pathway assignments	517
No. of antibiotic resistance genes	15 (*mecA*, *blaZ*, *ermC*, *msrA*, *mphC*, *vgaALC*, *AAC(6')-Ie-APH(2'')-Ia*, *mupA*, *norC*, *sdrM*, *sepA*, *qacJ*, *vanY*, *VanT*, and *gyrA*)
No. of virulence factor (VFs) genes	9

Closest related species	*Staphylococcus haemolyticus* strain B	*Staphylococcus haemolyticus* strain VB5326
GenBank accession no.	NZ_CP025396.1	NZ_CP045137.2
Query coverage (%)	94.16	92.93
Template coverage (%)	92.78	89.01

## Data Availability

The whole-genome shotgun (WGS) project of the *Staphylococcus haemolyticus* strain MRSH_GSTU_NS23_BD has been deposited in DDBJ/ENA/GenBank under accession number JBWQPX000000000. The version described in this paper is JBWQPX000000000.1. Associated data, including the raw sequencing reads, are available under BioProject accession number PRJNA1445815, BioSample accession number SAMN56799113, and SRA accession number SRR37897094.
